# Automatic Sampling and Analysis of Organics and Biomolecules by Capillary Action-Supported Contactless Atmospheric Pressure Ionization Mass Spectrometry

**DOI:** 10.1371/journal.pone.0066292

**Published:** 2013-06-06

**Authors:** Cheng-Huan Hsieh, Anil Kumar Meher, Yu-Chie Chen

**Affiliations:** Department of Applied Chemistry, National Chiao Tung University, Hsinchu, Taiwan; Imperial College London, United Kingdom

## Abstract

Contactless atmospheric pressure ionization (C-API) method has been recently developed for mass spectrometric analysis. A tapered capillary is used as both the sampling tube and spray emitter in C-API. No electric contact is required on the capillary tip during C-API mass spectrometric analysis. The simple design of the ionization method enables the automation of the C-API sampling system. In this study, we propose an automatic C-API sampling system consisting of a capillary (∼1 cm), an aluminium sample holder, and a movable XY stage for the mass spectrometric analysis of organics and biomolecules. The aluminium sample holder is controlled by the movable XY stage. The outlet of the C-API capillary is placed in front of the orifice of a mass spectrometer, whereas the sample well on the sample holder is moved underneath the capillary inlet. The sample droplet on the well can be readily infused into the C-API capillary through capillary action. When the sample solution reaches the capillary outlet, the sample spray is readily formed in the proximity of the mass spectrometer applied with a high electric field. The gas phase ions generated from the spray can be readily monitored by the mass spectrometer. We demonstrate that six samples can be analyzed in sequence within 3.5 min using this automatic C-API MS setup. Furthermore, the well containing the rinsing solvent is alternately arranged between the sample wells. Therefore, the C-API capillary could be readily flushed between runs. No carryover problems are observed during the analyses. The sample volume required for the C-API MS analysis is minimal, with less than 1 nL of the sample solution being sufficient for analysis. The feasibility of using this setup for quantitative analysis is also demonstrated.

## Introduction

The field of atmospheric pressure ionization (API) mass spectrometry has grown quickly since the inspired work of desorption electrospray ionization (DESI) was reported by Cooks and co-workers in 2004 [1]. Furthermore, because of the improved performance of mass spectrometers, the recent development of atmospheric pressure ion sources is striking. In the past few years, many new ion sources[1], [2], [3], [4], [5], [6], [7], [8], [9], [10], [11], [12], [13], [14],[15], [16], [17], [18] such as one-step[1], [6], [7], [8], [9], [10], [11], [12], [13], [14],[15] and two-step [16], [17], [18] ionization methods have been reported. The main advantages of these ionization methods over conventional API techniques are their simplicity and convenience through the elimination and reduction of sample preparation steps. Combined with suitable mass analyzers, qualitative information regarding molecular weights and analyte structures can be obtained. Automatic sampling API mass spectrometry provides the possibility for conducting quantitative analysis. Previously, automatic scanning probe electrospray ion source for imaging mass spectrometry has been proposed for the analysis of small molecules [15]. Automatic sampling atmospheric pressure chemical ionization (APCI) was recently proposed for quantitative analysis, but its setup requires several pumps and valves [19].

Additionally, chip-based nanoelectrospray equipped with silicon wafer with nozzles etched on the surface has been employed for auto-sampling analysis. However, sophisticated accessories are required in the setup [20], [21]. Additionally, laser diode thermal desorption APCI has been employed to high throughput analysis. A diode laser and an APCI source are required for the setup [22], [23]. High-throughput quantitation of antibiotics from diary milk using this technique has been demonstrated [23]. Nevertheless, employing APT techniques directly in quantitative analysis remains a challenge.

An API technique, called contactless API (C-API) [24], [25], [26], was developed by simply placing a tapered capillary as the sampling tube and spray emitter next to ∼1 mm from the orifice of a mass spectrometer to facilitate analyte ionization. Although no electrical connection or any external force is applied on the capillary, the sample solution is introduced from the capillary inlet to the outlet through capillary action. The eluent in the capillary outlet is polarized via induction with a high electric field provided by the mass spectrometer, which leads to charge accumulation that forms fine droplets after liquid disruption, and generates gas-phase ions after solvent evaporation [24], [25], [26]. The length and diameter of the C-API capillary vary depending on the requirement of the experimental setup. The C-API capillary can be 1 cm to 20 cm long [24], [25]. A longer capillary is suitable as a sampling tube and spray emitter in C-API MS, but the time required for introducing samples from the capillary inlet to the outlet is longer. On the other hand, the sampling volume can be controlled through the sampling time in the C-API approach. Furthermore, because capillary tubes are used as sampling tubes, the sample infusion volume can be as low as the subnanoliter level. Given the simple setup of C-API, an automatic sampling system can be readily established. A C-API–based automatic sampling system for multiple sample analyses is proposed and the feasibility of using this setup for quantitative analysis is discussed.

## Materials and Methods

All of the amino acids, caffeine, and creatinine were purchased from Sigma (St. Louis, MO, USA). Acetonitrile was purchased from Merck (Darmstadt, Germany). Fused silica capillary tubes [150 µm outer diameter (o.d.) and 10 µm inner diameter (i.d.)] were obtained from GL Science (Japan). Aluminium sheets (3 cm×3 cm×2 mm) and stainless steel tubes (0.8 mm o.d. and 0.5 mm i.d.) were obtained from local companies.

### Fabrication of C-API Capillary

A tapered capillary was used as the C-API sampling tube and spray emitter. The tapered capillaries were fabricated based on a previously described method [24], [25], [26]. A 50 g weight was attached to the lower end of a vertically positioned capillary. A propane/butane flame was used to heat the lower part of the capillaries. A narrow tip was then quickly created at the lower capillary part. After cooling to room temperature, the capillary tips were immersed in 24% (v/v) hydrofluoric acid solution for 30 min. The tip was then flushed with methanol and deionized water followed by conditioning with aqueous NaOH (1 M) and deionized water for 30 min using a pump. The tip was checked under an optical microscope before use. The diameter of the tapered tip was around 6–8 µm. A 1 cm tapered capillary section was cut and used as the C-API sampling tube and spray emitter.

### Design of C-API Automatic-Sampling Setup

**Figure 1:**
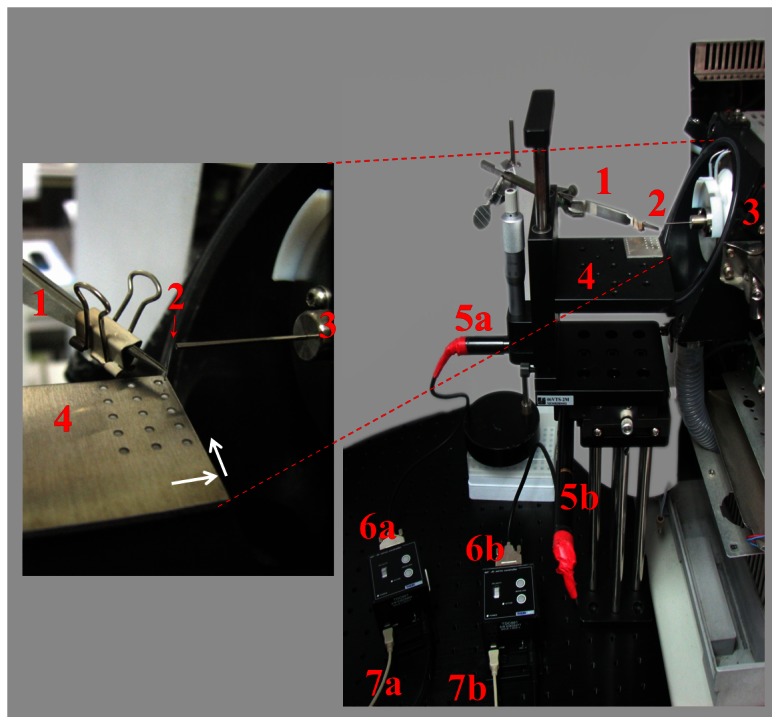
Photograph of the automatic-sampling C-API setup. 1, forceps; 2, 1 cm long capillary; 3, mass spectrometer; 4, XY movable stage; 5a and 5b, actuators; 6a and 6b, controllers; and 7a and 7b, cables to computers.

The tapered capillary (1 cm) prepared above was held using a forceps, which was fixed with a clamp and an adjustable stand (Fig. 1). An aluminium sheet (3 cm×3 cm×2 mm) was used as the sample holder, whereas small round sample wells (1 mm in diameter and 0.5 mm in depth) were fabricated using a SDPL-50W (Sintec Optronics Pte, Singapore) µs-laser engraving machine. The machine was equipped with a Nd:YAG diode-pumped solid-state laser (λ = 1064 nm) and controlled using the Laser Marker software (version 3.2.3.5, Jinan DuoWei Laser Technology, Jinan, Shandong, China). Each well (1 mm in diameter) on the aluminium sample holder had a 3 mm (center-to-center) distance between each well. The aluminium sample holder was placed on a motorized XY stage regulated by motorized actuators (Z825B, Thorlabs) and controllers (TDC001, Thorlabs) and controlled with the T-cube DC servo motor driver software. Sample droplets (4 µL) were deposited in each well prior to MS analysis. During C-API automatic sampling analysis, the C-API capillary positioned in front of the orifice of an Esquire 2k ion trap mass analyzer (Bruker Daltonics, Bremen, Germany), was initially immersed into a rinse solvent [acetonitrile/deionized water (1∶1, v/v)] to conduct automatically flush for 10 s (or 30 s). Simultaneously, the MS was activated and it continually acquired the MS data. After 10 s, the aluminium plate was programmed to move quickly in x-direction for 3 mm (or 4 mm) and to allow the C-API capillary to be immersed into the next sample droplet, which consisting of sample solution. The MS continued to acquire data to monitor the changes in the ion signals. After 10 s (or 20 s), the aluminium plate was then moved 3 mm further in the x-direction and allowed the C-API capillary to sample the next well of rinse solvent for flushing through capillary action. The steps were repeated as a cycle during the auto-sampling C-API analysis. The maximum moving speed was set to 3 mm s^−1^ (with an average acceleration of 1 mm s^−2^). All movements steps were programmed using the T-cube DC servo motor driver software.

## Results and Discussion

**Figure 2:**
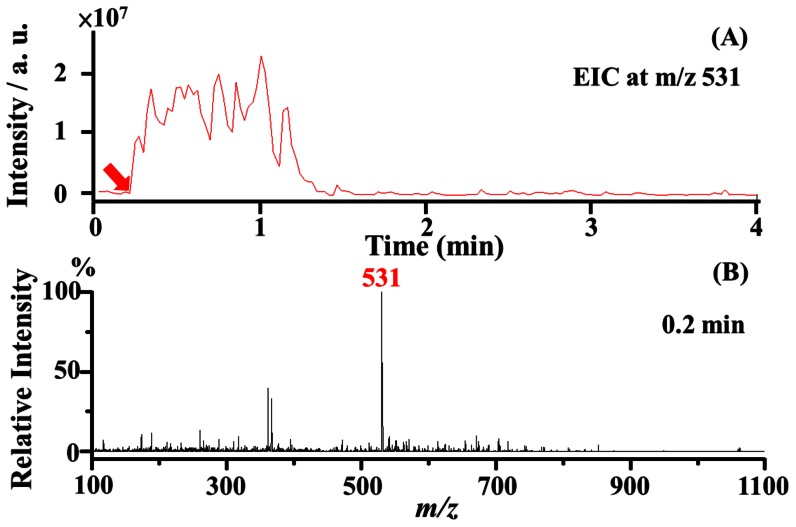
Flow rate examination. (A) The extracted ion chromatogram (EIC) at *m/z* 531, which corresponds to the doubly charged bradykinin ions, and (B) the corresponding mass spectrum obtained at the 0.2 min time point from panel A. The results were obtained using a capillary tapered to ∼7 µm (1 cm in length) as the C-API sampling tube and spray emitter. The capillary was filled with acetonitrile/deionized water (1∶1, v/v) prior to MS analysis. The C-API capillary was placed in front (∼1 mm) of a mass spectrometer and then immersed into a sample droplet containing 10 µM bradykinin prepared in acetonitrile/deionized water (1∶1, v/v). Upon immersion of the capillary into the sample droplet, the mass spectrometer was activated.

This study focused on the development of automatic sampling MS analysis using the C-API setup. Faster sampling speeds are helpful for high-throughput analysis. In principle, a thinner capillary tube would provide a faster sampling speed. Thus, a capillary (150 µm o.d., 10 µm i.d., 1 cm in length) was tapered and used as the sampling tube and the C-API spray emitter. Bradykinin (10 µM) in acetonitrile/deionized water (1∶1, v/v) was initially used as the model sample. The tapered capillary filled with acetonitrile/deionized water (1∶1, v/v) was placed in front of a mass spectrometer (Fig. 1). After a 4 µL sample droplet was deposited on the aluminium plate on a XY stage, the plate was moved up to immerse the capillary inlet into the small droplet (Fig. 1). Simultaneously, the mass spectrometer acquired the MS signal. The sampling speed was estimated based on the time of doubly charged bradykinin ion signal observed in the extracted ion chromatogram (EIC) at *m/z* 531. Figure 2A shows the EIC at *m/z* 531, whereas Figure 2B shows the mass spectrum obtained at the 0.2 min time point when the peak at *m/z* 531 just appeared. The flow rate was estimated to be ∼4 nL min^−1^, whereas the linear velocity was estimated to be ∼5 cm min^−1^. The results indicate that the thin capillary tube provided a fast sampling speed. However, capillary tips that were thinner than used herein were too fragile to be held with tweezers in the current design. Thus, the smallest capillary tube with an original i.d. of 10 µm was used as the C-API sampling tube and spray emitter in this study.

**Figure 3:**
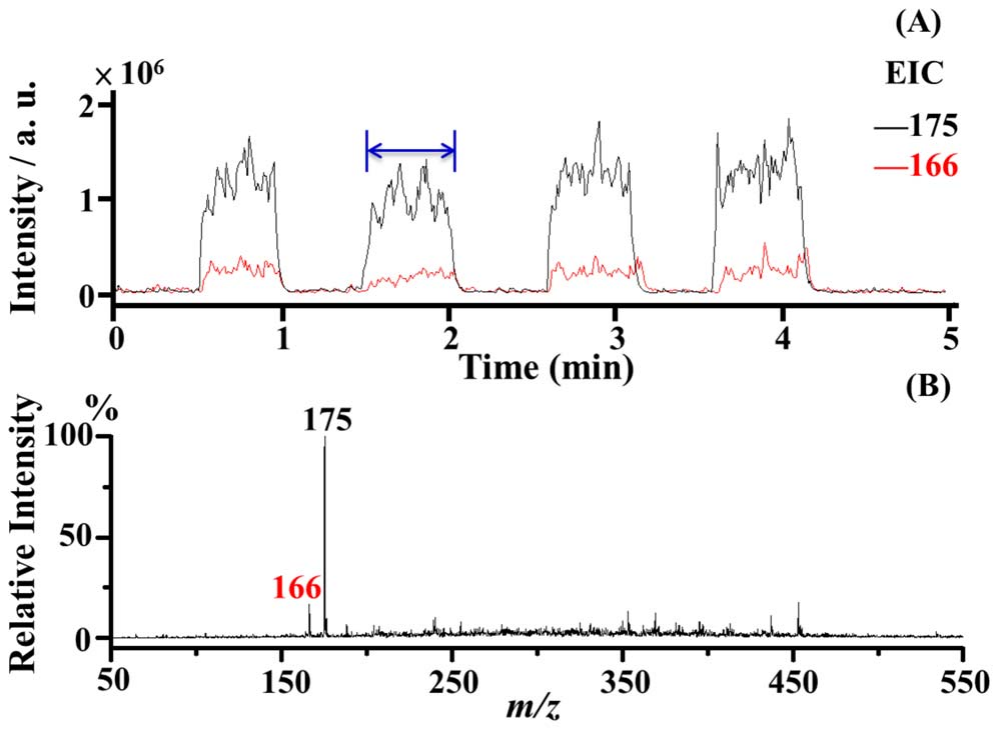
Examination of four replicates. A sample (4 µL) containing arginine (4 µM, MH^+^ = 175) and phenylalanine (1 µM, MH^+^ = 166) was as deposited in the sample well on the aluminium sample holder (yellow circles in the inset in panel A). The rinse solvent was alternately deposited next to the sample wells (white circles shown in the inset in panel A) for auto-flushing before every run. (A) The resultant EIC plots of the ions at *m/z* 175 (black plot) and at *m/z* 166 (red plot). (B) Corresponding mass spectra recorded from 1.5 min to 2 min in panel A.

To examine the feasibility of using the thin capillary as the sampling tube and the C-API spray emitter for autosampling operations, an aluminium plate was placed on a motorized XY stage (Fig. 1). Disorders of amino acid metabolism cause serious health problems [27]. Detection of amino acids in biological fluids is used in the diagnosis of inborn errors of metabolism in infancy. Thus, we selected amino acids as the model samples. Initially, the mixture of arginine (4 µM) and phenylalanine (1 µM) was used as the sample solution. The experiment was investigated by placing the sample solution with the same concentration in four wells on the aluminium sample plate. Every other sample well was loaded with rinse solvent [acetonitrile/deionized water (1∶1, v/v)] for rinsing the capillary after analysis. The inset in Figure 3A shows how the sample wells were arranged. The sampling capillary was flushed after every run. Surface tension allowed small sample droplets to form on the well upon sample deposition. When the sampling capillary was immersed into the liquid droplet, the sample solution is automatically introduced from the capillary inlet to the outlet through capillary action. The tube remained in the sample droplet for 10 s and ∼0.7 nL of sample solution was introduced into the sampling tube, with a flow rate of ∼4 nL min^−1^ as previously estimated. The plate was then moved again and allowed the sampling capillary to be immersed in the next well containing rinse solvent [acetonitrile/deionized water (1∶1, v/v)] for flushing through capillary action. After 20 s, the aluminium plate was quickly moved and allowed the capillary to be immersed in the next sample droplet for MS analysis. Figure 3A shows the EIC plot at *m/z* 175 (arginine, MH^+^) and *m/z* 166 (phenylalanine, MH^+^). The analyte signals lasted for ∼30 s in each run. After capillary flushing, the analyte ions disappeared (Fig. 3A). This finding shows that no carryover problems occurred during analysis. The average of the ratios of the peak area at *m/z* 175 to that at *m/z* 166 of four replicates was 5.03±0.31, whereas the relative standard deviation was 6.2%. Figure 3B shows the mass spectrum acquired from 1.5 min to 2 min. The peaks at *m/z* 166 and *m/z* 175 dominated the mass spectrum and no apparent background ions were observed in the same mass spectrum. Furthermore, Figure 3 shows that similar results from the same sample can be repeatedly obtained from different runs using the C-API autosampling system. Considering autowashing steps were also conducted between each run, no carryover problems were observed. Because no electric connection was applied on either the capillary inlet or outlet, the autosampling setup can be easily established.

**Figure 4:**
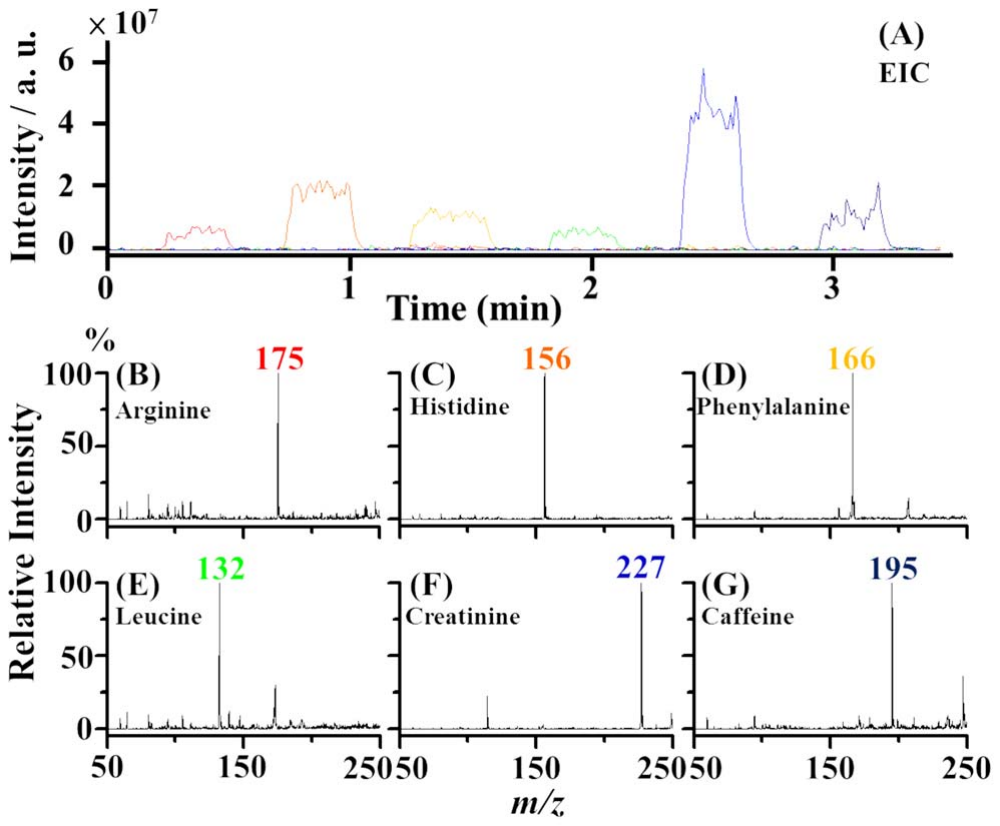
Multiple sample analysis. Four µL samples of arginine (5×10^−6^ M, MH^+^ = 175), histidine (5×10^−5^ M, MH^+^ = 156), phenylalanine (10^−4^ M, MH^+^ = 166), leucine (10^−4^ M, MH^+^ = 132), creatinine (10^−4^ M, M_2_H^+^ = 227), and caffeine (10^−4^ M, MH^+^ = 195) were deposited into different wells in two dimensions on the aluminium sample holder as shown in Figure 1. The rinse solvent [acetonitrile/deionized water (1∶1, v/v)] was loaded to alternate with the sample wells for autoflushing after every run. (A) The resultant EIC plots of the ions at *m/z* 175, 156, 166, 132, 227, and 195. Panels (B) to (G) show the mass spectra obtained at the time points of 0.2 min to 0.5 min, 0.7 min to 1.0 min, 1.3 min to 1.6 min, 1.8 min to 2.1 min, 2.4 min to 2.7 min, and 2.9 min to 3.2 min, respectively, in panel A.

The C-API autosampling system was also employed for multiple sample analyses to show the possibility of using the automatic system for high throughput analysis. Several standards were selected. In addition to amino acids as mentioned above, creatinine was selected as model sample. Creatinine is a common metabolite found in urine. Caffeine is also a common component present in drinks. The sample wells were arranged in two-dimensions on an aluminium plate, as shown in Figure 1. Arginine (MH^+^ = 175), histidine (MH^+^ = 156), phenylalanine (MH^+^ = 166), leucine (MH^+^ = 132), creatinine (M_2_H^+^ = 227), and caffeine (MH^+^ = 195) were selected as model samples and loaded onto the sample wells on the aluminium plate. The rinse solvent [acetonitrile/deionized water (1∶1, v/v)] was loaded alternately with the sample wells for sampling capillary flushing after every run. Figure 4A shows the EIC plots of the analyte ions at *m/z* 175, 156, 166, 132, 227, and 195, whereas the corresponding mass spectra obtained at the time points of 0.2 min to 0.5 min, 0.7 min to 1.0 min, 1.3 min to 1.6 min, 1.8 min to 2.1 min, 2.4 min to 2.7 min, and 2.9 min to 3.2 min, respectively, are shown in Figures 4B to 4G. All analyte ion peaks dominated their own mass spectra. Furthermore, no carryover effects were observed. The analysis using six samples was completed within 3.5 min, including six alternate flushings. The results demonstrated that the C-API autosampling system can be used for analyzing many samples within a short period. The flushings of the sampling capillary can be performed automatically and effectively after each run. Cross-contamination problems were not observed during analysis.

**Figure 5:**
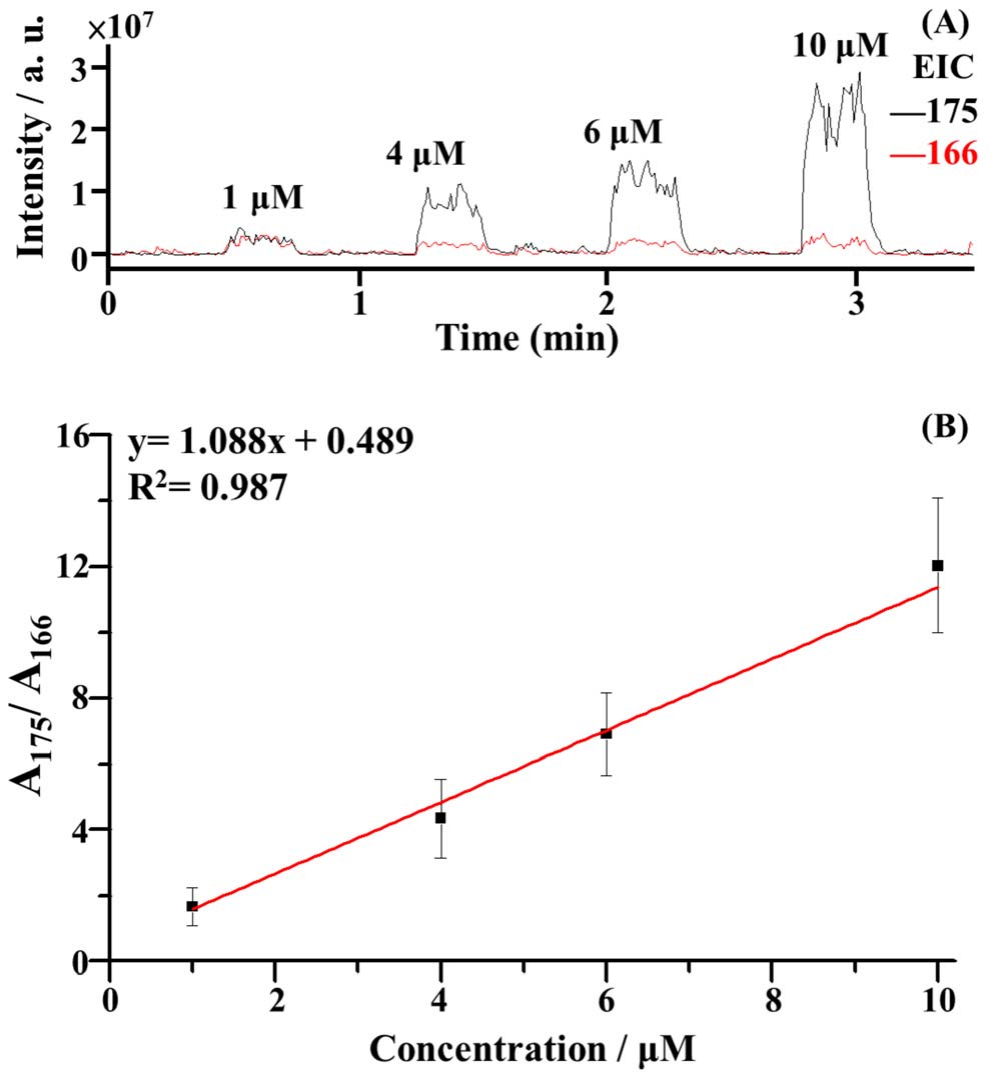
Calibration curve. (A) Representative EIC plots of the ions at *m/z* 175 (protonated arginine) and *m/z* 166 (protonated phenylalanine) obtained from a series of samples containing various arginine concentrations (1, 4, 6, and 10 µM) and phenylalanine (1 µM) as the internal standard for autosampling C-API MS analysis. Capillary flushing was conducted on every other well using acetonitrile/deionized water (1∶1, v/v) as the rinse solvent. (B) Plot obtained from the peak area ratio at *m/z* 175 to that at *m/z* 166 versus the arginine concentration as obtained from panel A. The results were obtained from three replicates using the same capillary as the sampling tube and C-API emitter.

The feasibility of using the C-API autosampling system for quantitative analysis was examined further. The aluminium plate was loaded with different arginine concentrations (1, 4, 6, and 10 µM), whereas phenylalanine (1 µM) was used as the internal standard. The rinse solvent (acetonitrile/deionized water (1∶1, v/v)) was placed in every other well next to the sample wells. Figure 5A shows the representative EIC plots at *m/z* 175 and 166. The peak area at *m/z* 175 increased as the arginine concentration in the sample increased. The peak area of the internal standard at *m/z* 166 did not change much. Figure 5B shows the plot of the ratio of the peak area at *m/z* 175 to that at *m/z* 166 versus the arginine concentration with an acceptable linear regression coefficient. The results demonstrated that the possibility of using the C-API auto-sampling system approach for quantitative analysis.

**Figure 6:**
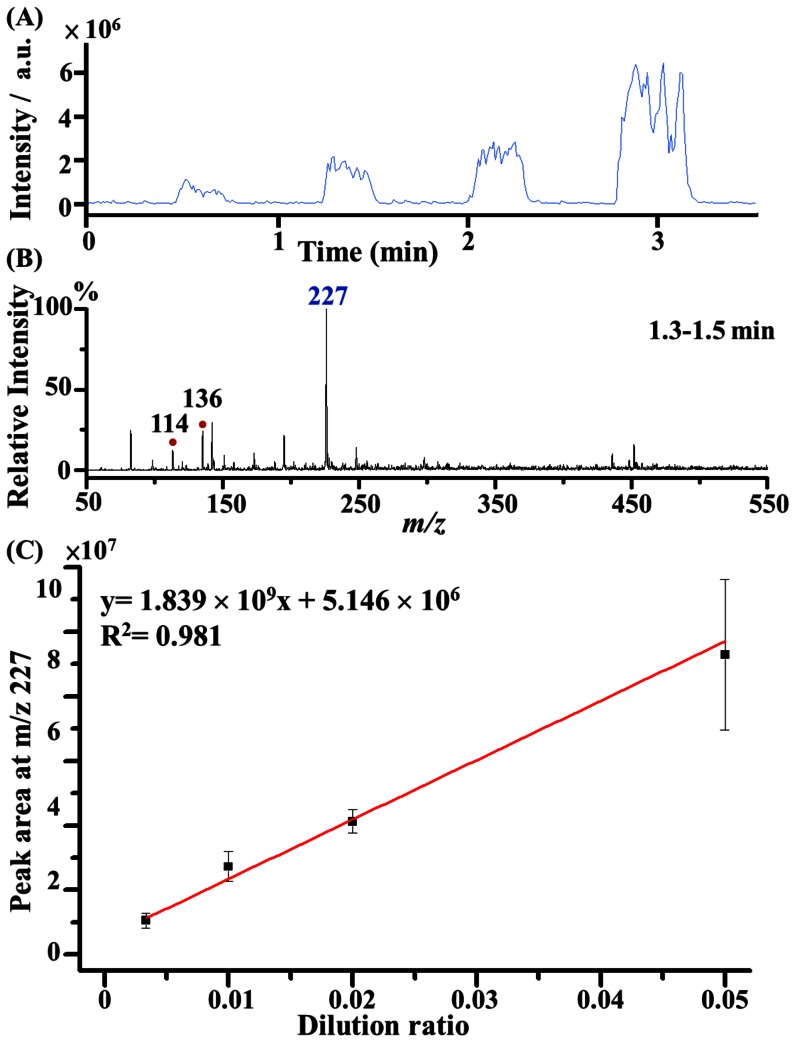
Analysis of creatinine in urine samples. (A) Representative EIC of the ion at *m/z* 227 obtained from a series of diluted urine samples by acetonitrile/deionized water (1∶1, v/v). (B) Corresponding mass spectrum acquired from 1.3 min to 1.5 min in panel A. (C) Plot of the peak area at *m/z* 227 versus the volume ratio of urine to the acetonitrile/deionized water (1∶1, v/v) solvent. The results were obtained from three replicates using the same capillary as the sampling tube and C-API emitter.

Additionally, the possibility of using biological fluids such as urine as sample was examined to investigate the feasibility of using the C-API auto-sampling system in real-life applications. Creatinine is one of important urine metabolites. It was chosen as the target analyte in the study. Urine with different dilution factors such as 20−, 50−, 100−, and 300−fold were utilized as samples for analysis. The change in the intensity of the creatinine signal and the correlation of the analyte ion signal with the dilution factor were determined. Considering the ion peak of protonated creatinine at *m/z* 114 overlapped with an unknown background peak, the ion peak of the most abundant creatinine dimer ion at *m/z* 227 was alternatively chosen as the target peak. Figure 6A shows the representative EIC plot at *m/z* 227. The peak area at *m/z* 227 increased as the urine became less dilute. When the aluminium plate was moved to the well with rinse solvent, no creatinine ions were observed in the EIC plot after capillary flushing. No carryover problems were observed during the analysis of the complex biological samples. Figure 6B shows the corresponding mass spectrum acquired between 1.3 min and 1.5 min. The peak at *m/z* 227 is the base peak, whereas the protonated creatinine ion at *m/z* 114 and the creatinine sodium adduct ion at *m/z* 136 appeared in the same mass spectrum. Figure 6C shows the plot of the peak area at *m/z* 227 versus the volume ratio of the urine to the solvent [acetonitrile/deionized water (1∶1, v/v)]. Although the urine samples contained a complex matrix, the complex matrix in the urine did not affect the quantitative analysis much in different dilution factors. An acceptable linear regression curve was obtained based on using the creatinine dimer ion as the target analyte to construct a calibration curve versus different urine dilutions.

**Figure 7:**
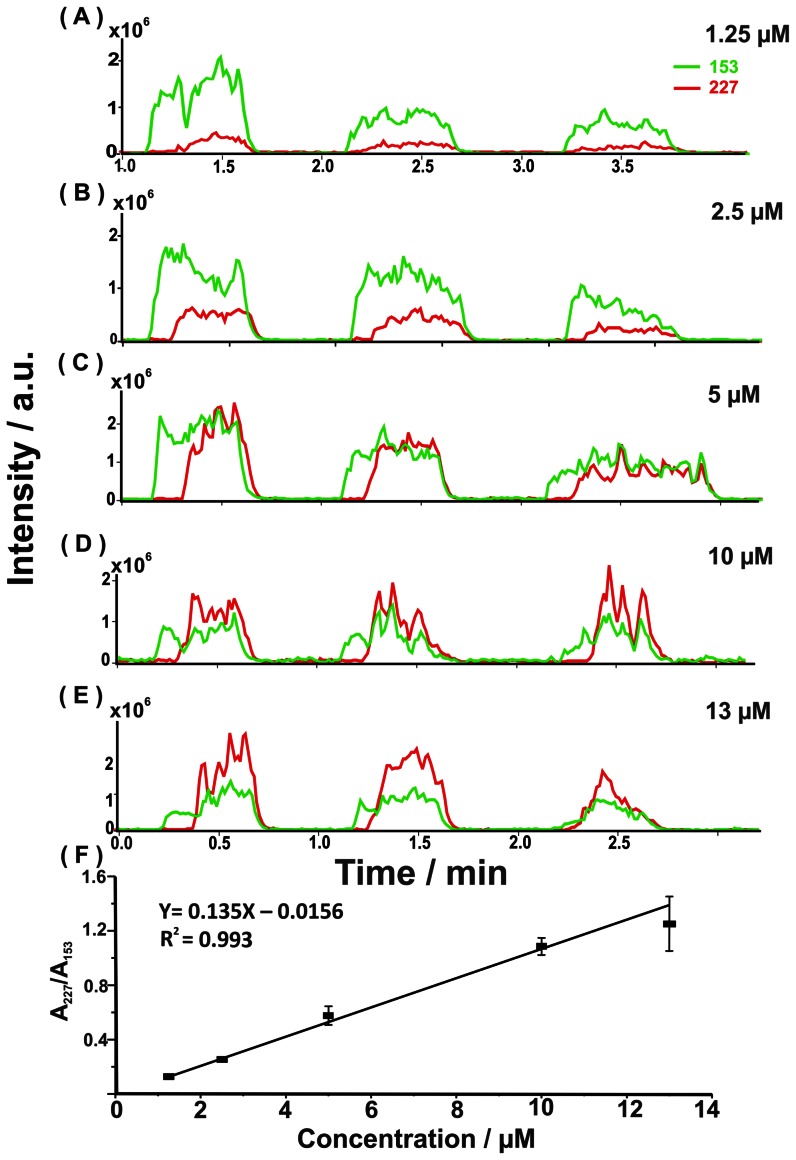
Calibration curve obtained from urine samples spiked with creatinine. (A) EICs of the ions at *m/z* 227 (red) and 153 (green) obtained from the 100-fold diluted urine samples spiked with creatinine with the concentrations of (A) 1.25×10^−6^ M, (B) 2.50×10^−6^ M, (C) 5.00×10^−6^ M, (D) 1.00×10^−5^ M, and (E), 1.30×10^−5^. Cystamine (10^−5^ M, MH^+^ = 153) was added to the urine samples as the internal standard (green curve). (F) Plot obtained from the ratio of the peak area at *m/z* 227 (A_227_) to the peak area at *m/z* 153 (A_153_) versus the concentration of creatinine spiked in the urine samples.

We also examined the result by spiking different concentrations of creatinine to 100-fold diluted urine. A given concentration of cystamine was spiked in the urine samples as the internal standard. The analysis of the samples containing different concentrations of creatinine spiked with the same concentration of cystamine (10^−5^ M) was repeated three times. Figures 7A-7E show the EIC plots at *m/z* 227 and 153 obtained from the 100-fold diluted urine samples spiked with different concentrations of creatinine and a given concentration of cystamine (10^−5^ M) for three replicates. The calibration curve was obtained using the current approach by plotting the ratio of the peak area of the peak at *m/z* 227 derived from creatinine dimer ions to the peak at *m/z* 153 derived from protonated cystamine ions (Fig. 7F). An acceptable linear calibration curve was obtained. The results indicated that using this current approach for quantitative analysis of complex samples is possible. Nevertheless, the setup should be further improved to reduce potential problems such as solvent evaporation during the analysis

## Conclusions

A simple automatic sampling C-API MS design has been demonstrated. Considering no external force is required in the sampling process, the setup is quite straightforward. The automatic sampling system was based on placing an aluminium sample holder on an XY moveable stage controlled by a computer. Multiple sample analyses and sampling capillary flushing can be readily conducted automatically after every run. Subnanoliter samples were sufficient for the analysis. The lowest concentration that the current approach could detect was at the attomole level. Faster sampling speeds can further benefit the real-time monitoring of chemical and biochemical reactions by reducing the delay. Sensitivity and sampling speed may be potentially improved using thinner and shorter capillaries as the C-API sampling tube and spray emitter, but the clotting problem arising in thinner capillaries because of the presence of undesirable species such as salts in the sample solution should be considered. We have demonstrated the possibility of using the current approach in quantitative analysis. However, it should be noticed that properly controlling the experimental condition such as humidity can reduce the evaporation of sample solutions during analysis. Spiking an internal standard to samples can compensate the evaporation problems. Still, the current setup may be further modified by placing the C-API tip in a chamber with humidity control to improve its analytical performance.
